# First Insights into the Genome Sequence of *Clostridium oryzae* DSM 28571, Isolated from the Soil of a Japanese Rice Field

**DOI:** 10.1128/genomeA.00539-17

**Published:** 2017-06-15

**Authors:** Anja Poehlein, Anna Lena Gippert, Maaike J. Bierenbroodspot, Rolf Daniel

**Affiliations:** a Genomic and Applied Microbiology & Göttingen Genomics Laboratory, Institute of Microbiology and Genetics, Georg-August University of Göttingen, Göttingen, Germany; b Applied Bioinformatics in Microbiology Course of the Microbiology and Biochemistry M.Sc./Ph.D. Program, Georg-August University of Göttingen, Göttingen, Germany

## Abstract

*Clostridium oryzae* was originally isolated from the soil of a Japanese rice field. *C. oryzae* represents a novel species within the genus *Clostridium* and is associated with anaerobic rice straw degradation. Here, we present the draft genome sequence of *C. oryzae* DSM 28571 (5.076 Mbp), containing 4,590 predicted protein-coding genes.

## GENOME ANNOUNCEMENT

*Clostridium oryzae* is an obligate anaerobic, Gram-positive, and spore-forming bacterium which was isolated from the soil of a Japanese rice field, using rice straw as the substrate (1). Phylogenetic analysis based on gyrase A protein sequences revealed that *C. oryzae* is closely related to some members of the genus *Clostridium sensu stricto* and *Clostridium acetobutylicum*. *C. oryzae* represents a novel species within the genus *Clostridium*, with strain KC3 (DSM 28571) as the type strain (1).

Chromosomal DNA was extracted applying the MasterPure complete DNA purification kit, according to the instructions of the manufacturer (Epicentre, Madison, WI, USA). Illumina paired-end seqeuncing libraries were prepared using the Nextera XT DNA library preparation kit, as recommended by the manufacturer (Illumina, San Diego, CA, USA). Sequencing of DNA was performed employing an Illumina MiSeq instrument and Illumina MiSeq reagent kit version 3, as described by the manufacturer. Trimming of reads using Trimmomatic version 0.36 (2) resulted in 2,856,526 paired-end reads. The assembly performed with the SPAdes genome assembler software version 3.10.0 (3) resulted in 176 contigs (>500 bp), with an average coverage of 135-fold. The obtained contigs were validated using QualiMap version 2.1 (4). The draft genome consists of a single chromosome (5.076 Mbp), with an overall G+C content of 33.1%. Automatic gene prediction was performed by using Prokka (5). The draft genome harbored 4,590 predicted protein-coding genes, including 3,514 genes with a predicted function and 1,076 genes coding for hypothetical proteins. In addition, 12 rRNA genes, 1 transfer-messenger RNA (tmRNA), and 69 tRNA genes were predicted. Interestingly, 3 repeat regions were found in the genome of *C. oryzae*. Two of these regions were in close proximity to a clustered regularly interspaced short palindromic repeat (CRISPR)-associated (Cas) gene cluster present in the genome. The third repeat region was associated with another CRISPR-Cas gene cluster identified in the genome.

In addition to *Bacteroidetes* and the *Alphaproteobacteria*, clostridial species are low-abundance components of rice soil bacterial communities. Rice straw mainly contains approximately 37% cellulose, 37% hemicellulose, and 15% lignin (6). *C. oryzae* cannot utilize lignin, cellulose, or hemicellulose, but it uses carbohydrates as growth substrates. One of the main substrates used by *C. oryzae* is soluble starch. We could identify a gene coding for a starch phosphorylase, which converts starch to α-d-glucose-6-phosphate (7). *C. oryzae* can also grow on cellobiose and melibiose. During the fermentation of cellobiose, *C. oryzae* produces lactate, butyrate, acetate, formate, H_2_, and CO_2_ (1). Correspondingly, 3 for butyrate production (*puuE*, *buk*, and *ptb*) and 4 genes for acetate synthesis (*paaK, thiH, ackA,* and *pta*) were detected in the genome sequence. *C. oryzae* has been described as positive for different enzymatic activities, including α-galactosidase, β-glucosidase, and α-arabinosidase activities (1). Correspondingly, genes were detected in the genome which were related to these enzyme types.

### Accession number(s).

The whole-genome shotgun project has been deposited at DDBJ/EMBL/GenBank under the accession number MZGV00000000. The version described here is the first version, MZGV01000000.
